# Mitochondrial DNA Instability in Cells Lacking Aconitase Correlates with Iron Citrate Toxicity

**DOI:** 10.1155/2013/493536

**Published:** 2013-08-26

**Authors:** Muhammad A. Farooq, Tammy M. Pracheil, Zhejun Dong, Fei Xiao, Zhengchang Liu

**Affiliations:** ^1^Department of Biological Sciences, University of New Orleans, New Orleans, LA 70148, USA; ^2^Key Laboratory of Geriatrics, Beijing Institute of Geriatrics, Beijing Hospital, Ministry of Health, Beijing 100730, China

## Abstract

Aconitase, the second enzyme of the tricarboxylic acid cycle encoded by *ACO1* in the budding yeast *Saccharomyces cerevisiae*, catalyzes the conversion of citrate to isocitrate. *aco1*Δ results in mitochondrial DNA (mtDNA) instability. It has been proposed that Aco1 binds to mtDNA and mediates its maintenance. Here we propose an alternative mechanism to account for mtDNA loss in *aco1*Δ mutant cells. We found that *aco1*Δ activated the RTG pathway, resulting in increased expression of genes encoding citrate synthase. By deleting *RTG1*, *RTG3*, or genes encoding citrate synthase, mtDNA instability was prevented in *aco1*Δ mutant cells. Increased activity of citrate synthase leads to iron accumulation in the mitochondria. Mutations in *MRS3* and *MRS4*, encoding two mitochondrial iron transporters, also prevented mtDNA loss due to *aco1*Δ. Mitochondria are the main source of superoxide radicals, which are converted to H_2_O_2_ through two superoxide dismutases, Sod1 and Sod2. H_2_O_2_ in turn reacts with Fe^2+^ to generate very active hydroxyl radicals. We found that loss of Sod1, but not Sod2, prevents mtDNA loss in *aco1*Δ mutant cells. We propose that mtDNA loss in *aco1*Δ mutant cells is caused by the activation of the RTG pathway and subsequent iron citrate accumulation and toxicity.

## 1. Introduction

Respiratory metabolism in eukaryotes requires proteins encoded in both the nuclear genome and the mitochondrial genome (mtDNA). Mitochondrial genomes generally encode a small number of proteins, many of which are involved in respiratory metabolism [1, 2]. Maintenance of mtDNA is important for cell growth and survival. Oxidative damage to mtDNA causes respiratory deficiency and human diseases [3–5]. In higher eukaryotes, how the mitochondrial genome is maintained and transmitted is not well understood. However, studies using the budding yeast *Saccharomyces cerevisiae* have generated an abundance of data on how its mitochondrial genome is maintained [6, 7]. Many nuclear-encoded proteins of diverse functions are required for mtDNA maintenance. When mtDNA is mutated, yeast cells form the so-called “petite” mutants. Yeast can be categorized as those with wild type (rho^+^ cells), extensively deleted (rho^−^ petites), or with complete loss of (rho^0^ petites) mtDNA. How mutations in nuclear genes cause defects in mtDNA maintenance is complex and often indirect. For example, mutations in the yeast homolog of frataxin, Yfh1, lead to iron overload in mitochondria, defects in maturation of proteins containing iron-sulfur clusters such as the TCA cycle enzyme aconitase, mtDNA instability and respiratory deficiency, and so forth [8–10]. Yeast studies have suggested that iron citrate toxicity may be responsible for *yfh1* mutant phenotypes [11, 12]. Respiratory metabolism generates reactive oxygen species such as superoxide radicals. Superoxide dismutases, Sod1 and Sod2, localized in the cytoplasm and mitochondria, respectively, are responsible for converting superoxide radicals to relatively harmless hydrogen peroxide [13], which can react with ferrous iron (Fe^2+^) to generate highly reactive hydroxyl radicals through the Fenton reaction. Hydrogen peroxide is detoxified by enzymes such as catalases, converting hydrogen peroxide to oxygen and water [14]. Mutations in yeast catalases and superoxide dismutase lead to oxidative damage and reduced resistance to oxidants [14–16].

Besides resulting in respiratory deficiency, mutations in TCA cycle enzyme encoding genes also lead to variable defects in mtDNA maintenance [17, 18]. The most severe phenotype is caused by mutations in the *ACO1* gene encoding aconitase, followed by the *IDH1* gene encoding a subunit of mitochondrial isocitrate dehydrogenase [19]. It has been proposed that Aco1 has a novel function in mediating mtDNA maintenance by directly binding mtDNA [20, 21]. Mutations in *ACO1* and *IDH1* share several growth defect phenotypes, which can be partially rescued by mutations in *CIT1*, encoding the mitochondrial isoform of citrate synthase [17]. Expression of *CIT1*, *ACO1*, *IDH1*, and *IDH2* is under dual control of two transcriptional regulatory complexes, Rtg1/3 and Hap2/3/4/5 [22]. In cells with reduced or defective respiratory functions, expression of these genes is under increased control of Rtg1/3. Rtg1 and Rtg3 are two basic helix-loop-helix transcription factors in the retrograde response pathway that mediates signaling from mitochondria to the nucleus [23]. Activation of Rtg1/3 requires a cytoplasmic protein, Rtg2, which has an N-terminal ATP binding domain in the Hsp70/actin/sugar kinase ATP binding domain superfamily [24]. The retrograde response pathway, also known as the RTG pathway, is activated in response to defects in mitochondrial respiratory function. Cit1, Aco1, and Idh1/2 promote synthesis of *α*-ketoglutarate, a precursor of glutamate, which is a potent repressor of the RTG pathway [23]. Mutations in *ACO1*, which lead to both a block in mitochondrial respiratory function and glutamate auxotrophy [25–27], therefore, likely activate the RTG pathway. However, it is not clear whether the *RTG* pathway contributes to the phenotypes of aco1 mutants.

Mutations in *RTG2* and *CIT2* have been reported to suppress mtDNA instability due to mutations in *YFH1* [12]. In this study, we provide an alternative model to account for mtDNA loss due to an *aco1Δ* mutation. We found that mutations in either *RTG* genes, genes encoding citrate synthases, genes encoding mitochondrial iron transporters, or *SOD1* suppress *aco1Δ*-induced mtDNA loss. Therefore, we propose that iron citrate toxicity contributes to *aco1Δ* mutant phenotypes.

## 2. Materials and Methods

### 2.1. Strains, Plasmids, Growth Media, and Growth Conditions

 Yeast strains and plasmids used in this study are listed in Tables 1 and 2, respectively. Yeast mutant strains were created by either direct transformation with gene knockout cassettes or through meiotic segregation analysis of heterozygous diploids. Mutations were confirmed by PCR-genotyping, standard genotyping based on selection markers, phenotypic analysis, and/or immunoblotting using antibody against Aco1. The BY4741 rho^0^ strain was generated by one passage of rho^+^ cells grown in YPD medium supplemented with 15 *μ*g/mL ethidium bromide. Yeast cells were grown in SD (0.67% yeast nitrogen base plus 2% dextrose), YNBCasD (SD medium plus 1% casamino acids), YNBcasR (0.67% yeast nitrogen base, 1% casamino acids, and 2% raffinose), YPD (1% yeast extract, 2% peptone, 2% dextrose), or YPEthanol (1% yeast extract, 2% peptone, 2% ethanol) medium at 30°C. When necessary, amino acids, adenine, and/or uracil were added to the growth medium at standard concentrations to cover auxotrophic requirements [28].

### 2.2. Yeast Transformation and *β*-Galactosidase Activity Assays

Plasmids were transformed into yeast strains using the high-efficiency lithium acetate-PEG method and *β*-galactosidase assays were carried out as described [28]. For each plasmid and strain combination, assays were conducted in duplicates, and independent experiments were carried out two times. Specific activity of *β*-galactosidase is expressed as nmols of *o*-nitrophenol generated from substrate *o*-nitrophenyl-*β*-D-galactoside per min per mg protein.

### 2.3. DAPI Staining of Nuclear and Mitochondrial DNA and FLuorescence Microscopy

DAPI (4′6,-diamidino-2-phenylindole) staining of nuclear and mitochondrial DNA was carried out as described [28]. Briefly, yeast strains were grown in liquid YPD or YNBcasD medium at 30°C overnight to *A*_600_ ~ 0.8. Cells were collected by centrifugation and treated with 1 *μ*g/mL DAPI in 95% ethanol for 30 min and cell pellets were washed with sterile water three times. DAPI-stained DNA molecules in fixed cells were observed by fluorescence microscopy using a Nikon Eclipse E800 microscope equipped with an HBO 100 W/2 mercury arc lamp, a Nikon Plan Fluor 100X objective lens, and epifluorescence with a Nikon UV-2E/C filter set (excitation 340–380 nm and emission 435–485 nm). Digital images were acquired with Photometrics Coolsnap fx CCD camera and Metamorph Imaging Software (Molecular Devices, Sunnyvale, CA) and processed using ImageJ (National Institutes of Health) and Adobe Photoshop (Mountain View, CA).

### 2.4. Citrate Analysis

 Cells were grown in 10 mL YPD medium overnight to ~OD_600_ 1.0. Cultures were chilled in ice-cold water for 22 min and cells were collected by centrifugation at 4°C. Cell pellets were then washed twice in chilled water. Citrate levels were determined using a citrate assay kit (BioVision, CA, USA). Cells were disrupted in 500 *μ*L Assay Buffer in the kit using glass beads method. Cell extract was clarified by centrifugation at 21,000 g at 4°C for 15 min. 20 uL cell extract was analyzed for protein concentration using Bradford assay and the rest of cell extract (~350) was deproteinized in Amicon Ultra 4 column (10 kDa cutoff). Deproteinized extract was analyzed for citrate levels according to the protocol provided by the manufacturer. Citrate levels in different strains were normalized by protein concentration of cellular extract prior to deproteinization. Citrate concentration in the wild-type strain was 7.97 nmols/mg proteins, which was arbitrarily set as 1 unit. Citrate concentration in the wild-type strain determined in this study is similar to ~1.1 nmols/10^7^ cells reported previously [12].

## 3. Results and Discussion

### 3.1. *aco1*Δ Activates the RTG Pathway

**Figure 1:**
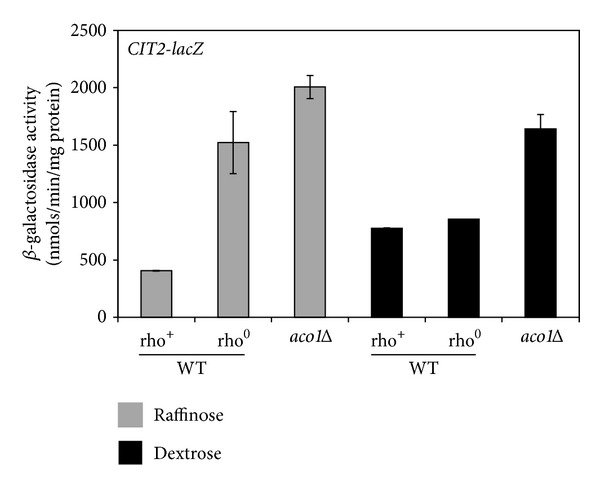
The RTG pathway is activated in *aco1Δ* mutant cells grown in dextrose medium. Wild-type rho^+^ (WT, BY4741), its rho^0^ derivative, and *aco1Δ* (ZLY2603) mutant strains were transformed with a centromeric plasmid encoding a *CIT2-lacZ* reporter gene (pCIT2-lacZ) and transformants were grown in YNBcasR (Raffinose) and YNBcasD (Dextrose) medium to mid-logarithmic phase. Cells were collected and *β*-galactosidase assays were conducted as described in Section 2.

Mutations in *ACO1* lead to both respiratory deficiency and glutamate starvation, which are expected to activate the RTG pathway. To test this possibility, we determined the effect of an *aco1Δ* mutation on the expression of a *CIT2-lacZ* reporter gene, which has been used extensively as a readout of the activity of the RTG pathway [22, 24, 33–35]. Expression of *CIT2-lacZ *was assessed in wild-type rho^+^ and *aco1Δ* mutant cells using *β*-galactosidase assays. Since *aco1Δ* cells are rho^0^ petites, we also determined *CIT2-lacZ* expression in otherwise wild-type rho^0^ cells. Cells were grown in rich media with either raffinose or dextrose (D-glucose) as the sole carbon source, which have been used in studies on the *RTG* pathway and mitochondrial genome maintenance, respectively [12, 20, 21, 36]. In cells grown in raffinose medium, *CIT2-lacZ* expression was 4-fold higher in rho^0^ cells compared to rho^+^ cells, consistent with previous reports that the *RTG* pathway is activated in rho^0^ cells [33, 36]. An *aco1Δ* mutation induced *CIT2-lacZ* expression slightly higher than in wild-type rho^0^ cells (Figure 1). In cells grown in dextrose medium, *CIT2-lacZ* expression in rho^0^ cells is only marginally higher than in rho^+^ cells, whereas an *aco1Δ* mutation almost doubled *CIT2-lacZ* expression. Activation of the RTG pathway is strain dependent [37]. The lack of induction of the RTG pathway due to loss of mtDNA in dextrose-grown BY4741 background strains used in this study can be partly attributed to a doubling of *CIT2-lacZ* expression in rho^+^ cells grown in this medium compared to raffinose medium, which is consistent with activation of the RTG pathway due to compromised mitochondrial respiratory function since dextrose suppresses respiratory metabolism in yeast. Altogether, these data indicate that an *aco1Δ* mutation leads to activation of the RTG pathway.

### 3.2. Mutations in RTG Genes Prevent mtDNA Loss due to *aco1Δ*

**Figure 2:**
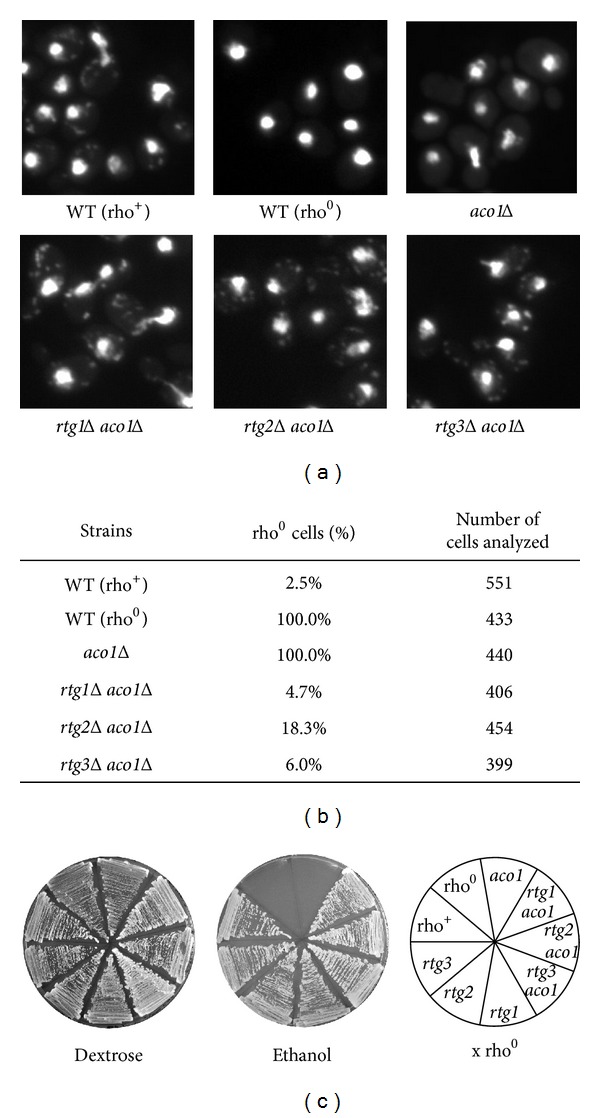
mtDNA is maintained in *rtg1Δ aco1, rtg2Δ aco1Δ, and rtg3Δ aco1Δ *mutant cells. (a) Cells grown exponentially in YPD medium were stained with DAPI and then examined using fluorescence microscopy. WT (rho^+^), BY4741; WT (rho^0^), BY4741 rho^0^; *aco1Δ*, ZLY2630; *rtg1Δ aco1Δ*, ZLY2648; *rtg2Δ aco1Δ*, ZLY2631; *rtg3Δ aco1Δ*, ZLY2652. (b) Quantitative analysis of the percentage of rho^0^ cells in yeast strains based on DAPI-staining images. (c) mtDNA in *rtgΔ aco1Δ* double mutant cells is functional. Strains as described in (a) as well as *rtg1Δ*, *rtg2Δ*, and *rtg3Δ* single mutants were crossed to a rho^0^ tester strain (X rho^0^) of opposite mating type. Diploids were selected and grown on YPD (Dextrose) and YPEthanol (Ethanol) medium. Pictures of cells were taken after 3 days' growth at 30°C.

 To determine whether activation of the RTG pathway in *aco1*Δ mutant cells contributes to mtDNA loss, *rtg1Δ aco1Δ*, *rtg2Δ aco1Δ*, and* rtg3Δ aco1Δ* double mutants were constructed and examined for the presence or absence of mtDNA. These double mutant strains were created by crossing respective haploid mutant strains to form heterozygous diploid mutants, which were then sporulated to generate desired haploid segregants. Seven *rtg1Δ aco1Δ*, seven *rtg2Δ aco1Δ*, and six *rtg3Δ aco1Δ* double mutant segregants were obtained. Eight *aco1*Δ single mutant segregants were also isolated similarly. DAPI, a DNA-specific probe that forms a fluorescent complex [38], was then used to visualize mtDNA using fluorescence microscopy in these mutants along with wild-type rho^+^ and rho^0^ strains. In addition to nuclear DNA, DAPI staining revealed punctate cytoplasmic structures of mtDNA in wild-type rho^+^ cells grown in YPD medium (Figure 2(a)) [38]. In contrast, mtDNA was absent in both wild-type rho^0^ and *aco1Δ* mutant cells, consistent with previous reports that Aco1 is required for mtDNA maintenance [17, 18, 21]. Interestingly, mtDNA was maintained in *rtg1Δ aco1Δ*, *rtg2Δ aco1Δ*, and *rtg3Δ aco1Δ* double mutant strains, indicating that Rtg proteins mediate mtDNA instability in *aco1Δ* mutant cells. The percentage of rho^0^ cells was quantified from DAPI-stained images and a large majority of *rtgΔ aco1Δ* double mutant cells were found to contain mtDNA (Figure 2(b)). Among seven *rtg2Δ aco1Δ* double mutant segregants from heterozygous *rtg2Δ/RTG2 aco1Δ/ACO1* diploid mutant cells, all were found to maintain mtDNA. Similarly, all of the seven *rtg1Δ aco1* and six *rtg3Δ aco1Δ* double mutant segregants were also found to be rho^+^ cells. In contrast, all of the eight *aco1Δ* single mutant segregants from a heterozygous *aco1Δ/ACO1* diploid mutant have lost mtDNA. Together, our data suggest that mtDNA instability in *aco1Δ* mutant cells may result from activation of the RTG pathway.

Damages to mtDNA can lead to extensive deletions (rho^−^) or point mutations (*mit* ^−^) [6, 7]. Yeast strains that carry these two types of mutant mitochondrial genomes are respiratory deficient. To determine whether the mtDNA in the *rtgΔ aco1Δ* double mutant strains is functional, we conducted a complementation assay by crossing wild-type rho^+^, wild-type rho^0^, *rtg1Δ*, *rtg2Δ*, *rtg3Δ*, *rtg1Δ aco1Δ*, *rtg2Δ aco1Δ*, and *rtg3Δ aco1Δ* mutant strains to a rho^0^ tester strain of opposite mating type with wild-type nuclear *ACO1* gene and analyzing the respiratory capacity of the resultant diploid strains. *rtg1Δ aco1Δ*, *rtg2Δ aco1Δ*, and *rtg3Δ aco1Δ* mutant strains were unable to utilize carbon sources that require respiratory metabolism such as ethanol because they are defective in the TCA cycle (data not shown), and Figure 2(c) shows that diploids generated from crossing wild-type rho^+^, *rtg1Δ*, *rtg2Δ*, and *rtg3Δ* strains with the rho^0^ tester strain were able to grow on ethanol medium. In contrast, diploids from crosses involving wild-type rho^0^ or the *aco1Δ* single mutant were unable to grow on ethanol medium, consistent with the absence of mtDNA in these diploids. Remarkably, diploids generated from the rho^0^ tester strain and *rtgΔ aco1Δ* double mutants were able to grow on ethanol medium, indicating that mtDNA in *rtgΔ aco1Δ* cells are functional.

### 3.3. Mutations in Genes Encoding Citrate Synthase, Primarily CIT1, Prevent mtDNA Loss due to *aco1Δ*

**Figure 3:**
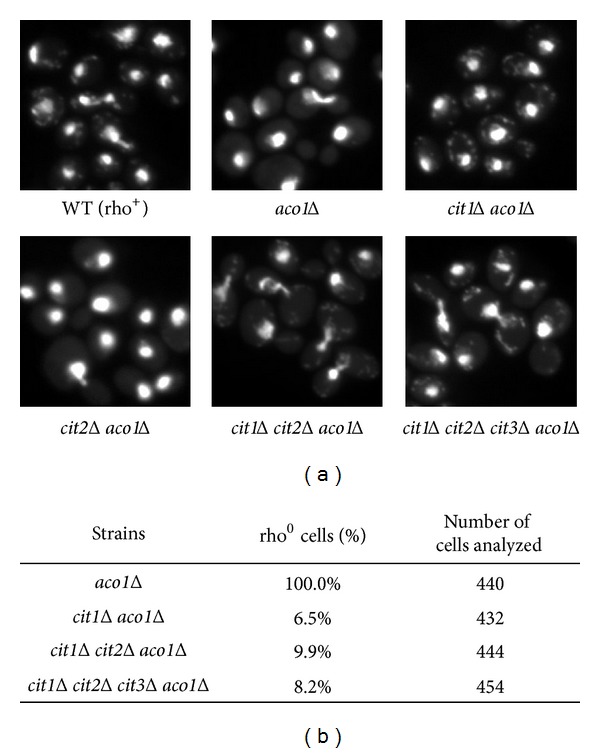
Mutations in genes encoding citrate synthase prevent mtDNA loss due to *aco1Δ*. (a) Cells grown in YPD medium to mid-logarithmic phase were stained with DAPI and examined using fluorescence microscopy. WT (rho^+^), BY4741; *aco1Δ*, ZLY2630; *cit1Δ aco1Δ*, RBY469; *cit2Δ aco1Δ*, RBY277; *cit1Δ cit2Δ aco1Δ*, ZLY854; *cit1Δ cit2Δ cit3Δ aco1Δ*, ZLY854; *rtg2Δ aco1Δ*, ZLY2545. (b) Quantitative analysis of the percentage of rho^0^ cells in yeast strains based on DAPI-staining images.

 What is the mechanism of *aco1Δ* suppression by mutations in *RTG* genes? The *RTG* pathway is required for glutamate biosynthesis in cells with reduced respiratory function by regulating expression of *CIT1*, *CIT2*, *ACO1*, *IDH1*, and *IDH2 *[23]. It has been shown previously that a *cit1Δ* mutation partially suppresses mtDNA loss in *aco1Δ* mutant cells and that mutations in *CIT2* and *RTG2* rescue respiratory deficiency in *yfh1Δ* mutant cells [12, 17]. Three genes, *CIT1*, *CIT2*, and *CIT3*, encode citrate synthase in yeast, with *CIT1* and *CIT3* encoding the two mitochondrial isoforms and *CIT2* encoding the peroxisomal isoform [39–41]. Therefore, suppression of mtDNA loss in *rtg1Δ aco1Δ*, *rtg2Δ aco1Δ*, and *rtg3Δ aco1Δ* double mutant cells may be due to reduced expression of genes encoding citrate synthase. To confirm this possibility, we introduced an *aco1Δ* mutation into a *cit1Δ cit2Δ cit3Δ* triple mutant in the BY4741 strain background generated by Chen et al. [21]. The presence or absence of mtDNA in the resultant quadruple mutant cells was examined by DAPI staining and fluorescence microscopy.  Figure 3(a) shows that the *cit1Δ cit2Δ cit3Δ aco1Δ* quadruple mutant maintained mtDNA. To determine which citrate synthase-encoding gene(s) is responsible for mtDNA loss in *aco1Δ* mutant cells, we generated *cit1Δ aco1Δ*, *cit2Δ aco1Δ*, and *cit3Δ aco1Δ* double mutants, as well as an *aco1Δ cit1Δ cit2Δ* triple mutant by crossing respective haploid mutant strains followed by meiotic segregation analysis. Using DAPI staining and fluorescence microscopy, we found that six out of eight *cit1Δ aco1Δ* double mutants, zero out of six *cit2Δ aco1Δ* double mutants, zero out of six *cit3Δ aco1Δ* double mutants, and six out of six *cit1Δ cit2Δ aco1Δ* triple mutant strains maintained mtDNA (Figure 3 and data not shown). We also calculated the percentage of rho^0^ cells in a *cit1Δ aco1Δ* double, a *cit1Δ cit2Δ aco1Δ* triple, and a *cit1Δ cit2Δ cit3Δ aco1Δ* quadruple mutant and found that over 90% cells from these mutants maintained mtDNA (Figure 3(b)). Taken together, these data suggest that citrate synthase is the target of the RTG pathway that mediates mtDNA instability in *aco1Δ* mutant cells and that Cit1 is primarily responsible for this phenotype in the BY4741 strain background.

### 3.4. Mutations in Genes Encoding Mitochondrial Iron Transporters *Mrs3* and *Mrs4* Prevent mtDNA Loss due to *aco1Δ*

**Figure 4:**
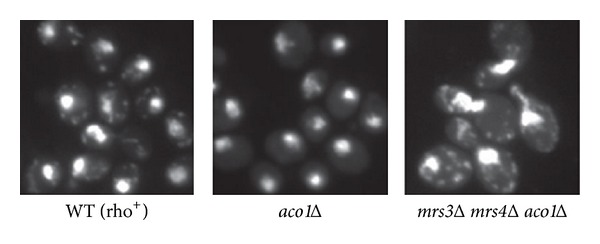
Mutations in *MRS3* and *MRS4*, encoding mitochondrial iron transporters, prevent mtDNA loss due to *aco1Δ*. Cells grown in YPD medium to mid-logarithmic phase were stained with DAPI and examined using fluorescence microscopy. WT (rho^+^), BY4741; *aco1Δ*, ZLY2630; *mrs3Δ mrs4Δ aco1Δ*, ZLY3505.

It has been proposed that iron citrate toxicity contributes to oxidative damage and mtDNA loss in *yfh1Δ* mutant cells, which have higher levels of cellular and mitochondrial iron [8, 12]. Mutations in *RTG2* and *CIT2* reduce petite formation in *yfh1Δ* mutants by lowering cellular citrate and iron levels. Suppression of mtDNA loss in *aco1Δ* mutant cells by mutations in *RTG* genes and genes encoding citrate synthase prompted us to test whether mitochondrial iron overload is responsible for mtDNA instability in *aco1Δ* mutants. Mitochondrial iron transport is mediated by iron transporters Mrs3 and Mrs4 [11, 29, 42, 43], mutations of which rescue mtDNA loss in *yfh1Δ* mutants. To this end, we generated an *mrs3Δ mrs4Δ aco1Δ* triple mutant by introducing an *aco1Δ* mutation into an *mrs3Δ mrs4Δ* double mutant. DAPI staining of the triple mutant showed that mtDNA was maintained (Figure 4). Furthermore, quantitative analysis showed that the percentage of rho^0^ cells in the triple mutant was 0.4%, slightly lower than 2.5% in wild-type rho^+^ cells. The *mrs3Δ mrs4Δ aco1Δ* triple mutant was also mated to a rho^0^ tester strain and the resulting diploids were streaked onto plates with ethanol as the sole carbon source. We found that the diploids could grow on ethanol medium, indicating that mtDNA in the *mrs3Δ mrs4Δ aco1Δ* triple mutant is functional (data not shown). Together, this data supports the notion that mtDNA loss in *aco1Δ* mutant cells is due to iron citrate toxicity.

It has been reported that the supplementation of exogenous iron (1 mM FeSO_4_) or raising pH of the growth medium reduces petite frequency in *aco1Δ* mutant cells grown in YPGalactose medium [17]. Thus, Lin et al. proposed that some of the effects of elevated citrate levels in *aco1Δ* are due to the ionized form of this metabolite rather than to the formation of a citrate/iron chelate that could produce oxidative damage to mtDNA. These observations seem to contradict our conclusion. However, iron homeostasis is a highly complicated process [44]. It was not established that exogenous iron actually increased iron levels in the mitochondria in Lin et al.'s study. It is also possible that growth conditions (YPD in this study versus YPGalactose in Lin et al.'s study) and/or strain backgrounds (BY4741 versus W303-1B) may affect mtDNA loss associated with *aco1Δ* mutations.

### 3.5. Iron Citrate Toxicity Correlates with mtDNA Loss in *aco1*Δ Mutant Cells

**Figure 5:**
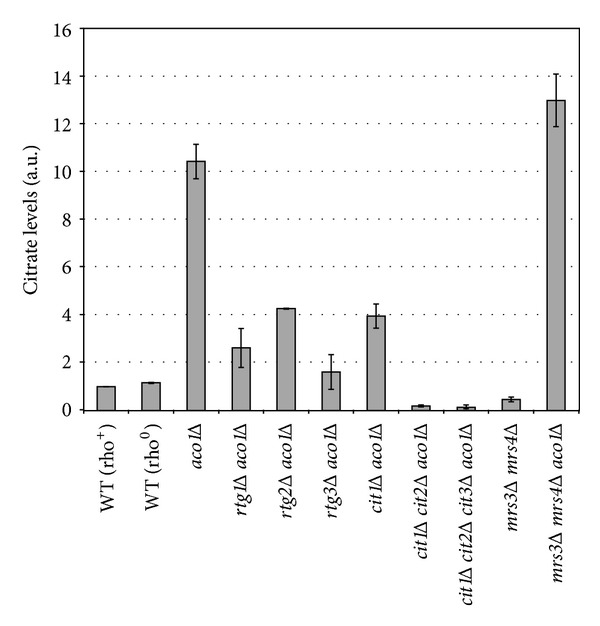
Citrate levels in wild-type (WT) and indicated mutant strains. Citrate concentration was determined as described in Section 2. The results are the average of two independent experiments.

 To affirm our hypothesis that iron citrate toxicity contributes to mtDNA loss in *aco1Δ* mutant cells, we determined citrate levels in wild-type and various mutant strains and found that *aco1Δ* increased the citrate level by 10.5-fold (Figure 5), which is consistent with an 11.9-fold increase reported by Lin et al. [17]. This increase is not due to that *aco1Δ* mutants are rho^0^ cells since the citrate level in wild-type rho^0^ cells was only 16% higher than that of wild-type rho^+^ cells. Mutations in *RTG1*, *RTG2*, and *RTG3* reduced citrate levels in *aco1Δ* background cells by 59–85%. Similarly, *cit1Δ* reduced citrate concentration by 62% in *aco1Δ* background cells, which is comparable to an 89% decrease reported by Lin et al. A double mutation in *CIT1* and *CIT2* reduced citrate concentration by 98% in *aco1Δ* background cells, and an additional mutation in *CIT3* did not significantly further reduce citrate levels. Clearly, there is strong correlation between the suppression of mtDNA loss phenotype and lower citrate levels in *aco1Δ* background strains. We also determined the effect of an *mrs3Δ mrs4Δ* double mutation on citrate levels. In comparison to a wild-type rho^+^ strain, citrate concentration in an *mrs3Δ mrs4Δ* double mutant was 53% lower. In contrast, citrate concentration in an *mrs3Δ mrs4Δ aco1Δ* triple mutant is 24% higher than that of an *aco1Δ* mutant. Since *mrs3Δ mrs4Δ aco1Δ* mutant cells maintained mtDNA, we propose that high levels of citrate per se are not sufficient to lead to mtDNA loss and that citrate toxicity requires certain levels of iron in the mitochondria. Together with our genetic data, these biochemical results suggest that iron citrate toxicity accounts for mtDNA loss in *aco1Δ* mutant cells.

### 3.6. A Mutation in SOD1, but Not *SOD2*, Prevents mtDNA Loss due to *aco1Δ*

**Figure 6:**
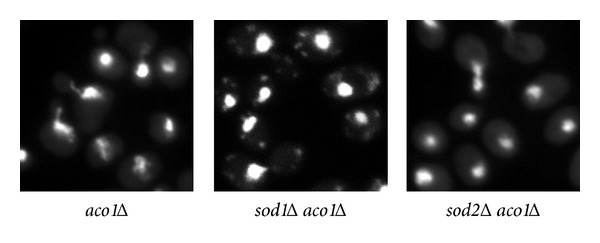
A mutation in *SOD1*, but not *SOD2*, suppresses mtDNA loss due to *aco1Δ*. Cells grown in YPD medium to mid-logarithmic phase were stained with DAPI and examined using fluorescence microscopy. *aco1Δ*, ZLY2630; *sod1Δ aco1Δ*, TPY1507; *sod2Δ aco1Δ*, ZLY3973.

Our data suggest that high levels of citrate cause mtDNA instability in *aco1Δ* mutant cells likely due to iron citrate toxicity. Iron reacts with hydrogen peroxide in the Fenton reaction to produce highly active, potent hydroxyl radicals, which cause oxidative damage to mitochondria. Hydrogen peroxide is partly produced by the superoxide dismutases, Sod1 in the cytoplasm and Sod2 in the mitochondrial matrix [12]. We hypothesized that a reduced production of hydrogen peroxide due to mutations in *SOD1* or *SOD2* might suppress mtDNA loss in *aco1Δ* mutant cells by lowering the amount of hydroxyl radicals produced via the Fenton reaction. Therefore, we generated *sod1Δ aco1Δ* and *sod2Δ aco1Δ* double mutants by crossing respective haploid mutant strains followed by meiotic segregation analysis. The resultant double mutant strains were analyzed for mtDNA presence by DAPI staining and fluorescence microscopy. 33 out of 34 *sod1Δ aco1Δ* double mutant strains generated maintained mtDNA while 6 out of 6 *sod2Δ aco1Δ* double mutants lost mtDNA (Figure 6 and data not shown). These data suggest that hydrogen peroxide generated from reactions catalyzed by Sod1 contributes to mtDNA loss in *aco1Δ* mutant cells. Mutations in *SOD1* also cause oxidative damage due to accumulation of superoxide radicals [15, 16]. However, since an *sod1Δ* mutation suppressed mtDNA loss in *aco1Δ* mutant cells, we propose that hydroxyl radicals are more damaging to mtDNA than superoxide radicals. How would mutations in the cytosolic isoform of superoxide dismutase rescue a mitochondrial defect? It has been shown that a small fraction of Sod1 is localized in the intermembrane space of mitochondria, which protects cells from mitochondrial oxidative damage [45–47]. Why does not *sod2Δ* suppress mtDNA loss associated with *aco1Δ*? It is possible that loss of Sod2 leads to increased levels of superoxide radicals in the mitochondria, which in the presence of ferrous ions would cause any hydrogen peroxide produced in the mitochondria to be converted to the hydroxyl radicals via the Fenton/Haber Weiss reactions that damage mtDNA.

### 3.7. Loss of mtDNA in *aco1Δ* Mutant Cells Is Growth Medium Dependent

**Figure 7:**
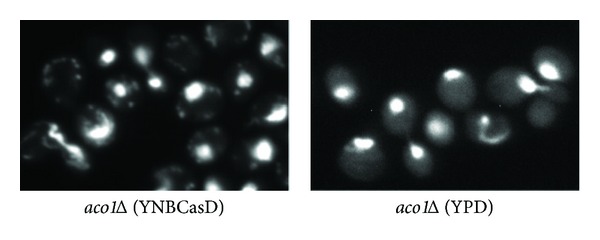
mtDNA loss due to *aco1Δ* is growth medium dependent. *aco1Δ* mutant cells (ZLY2630) carrying empty pRS416 vector were grown in YNBcasD or YPD medium to mid-logarithmic phase, stained with DAPI, and examined using fluorescence microscopy.

It has been proposed that mtDNA loss in *aco1Δ* mutant cells is due to lack of physical protection of mtDNA by Aco1 [20]. One key piece of evidence that supports this hypothesis is the observation that the expression of two catalytically inactive Aco1 mutants, Aco1^C382S^ and Aco1^C445S^, under the control of the *ADH1* promoter from the pRS416 centromeric plasmid, prevented mtDNA loss in *aco1Δ* mutant cells. To maintain the plasmids, transformants were grown in YNBcasD medium. In light of discovery that iron citrate toxicity contributes to mtDNA loss, one alternative explanation for mtDNA retention in cells expressing *ACO1* ^C382S^ and *ACO1* ^C445S^ mutant alleles is due to differences in growth medium, YNBcasD versus YPD. Accordingly, we transformed *aco1Δ/ACO1* heterozygous diploid mutant cells with empty pRS416 vector and transformants were sporulated and dissected on YPD or YNBcasD medium. Eleven *aco1Δ* haploid mutants from a YPD dissection plate were obtained and grown in YPD liquid medium and mtDNA was observed by DAPI staining. We found that all of the eleven *aco1Δ* segregants lost mitochondrial DNA (Figure 7 and data not shown). In contrast, among nine *aco1Δ* mutant segregants carrying the empty pRS416 vector from a YNBcasD dissection plate that were grown in YNBcasD liquid medium, seven maintained mtDNA (Figure 7 and data not shown). When these seven *aco1Δ* mutants containing mtDNA were passed onto YPD plate medium twice and then grown in YPD liquid medium, all lost mtDNA (data not shown). Together, these data suggest that mtDNA loss in *aco1Δ* mutant cells is growth medium dependent.

## 4. Conclusions

It has been proposed that yeast aconitase (Aco1) physically binds to mtDNA and promotes its maintenance [20, 21]. Our results in this study suggest a different, but not necessarily mutually exclusive, mechanism. We propose that *aco1Δ* activates the RTG pathway, resulting in increased citrate production through upregulation of genes encoding citrate synthase. Increased levels of citrate lead to iron overload in the mitochondria. Iron then reacts with hydrogen peroxide to generate hydroxyl radicals, which cause oxidative damage to mitochondrial DNA and consequently its instability. Mutations of yeast frataxin (Yfh1) lead to loss of activity of aconitase [48]. Suppression of mtDNA instability due to mutations in both *YFH1* and *ACO1* by reduced iron citrate levels raises the possibility that mtDNA loss in *yfh1* mutant cells may be an indirect consequence of aconitase inactivation. Our data also suggest that the cytosolic superoxide dismutase, Sod1, but not the mitochondrial superoxide dismutase, Sod2, contributes to mtDNA loss in *aco1Δ* mutant cells.

## Figures and Tables

**Table 1:** Strains used in this study.

Strain	Genotype	Source
BY4741	*MATa ura3 leu2 his3 met15 *	Research genetics
BY4731	*MATa ura3 leu2 met15 *	Research genetics
BY4742	*MATa ura3 leu2 his3 lys2 *	Research genetics
BY4741 (rho^0^)	BY4741 rho^0^ derivative	This study
ZLY2630	BY4741 *aco1::kanMX4 *	This study
ZLY1264	BY4741 *rtg1::kanMX4 *	Research genetics
ZLY1267	BY4741 *rtg2::kanMX4 *	Research genetics
ZLY1273	BY4741 *rtg3::kanMX4 *	Research genetics
ZLY2568	BY4742 *rtg1::LEU2 *	This study
ZLY143	BY4731 *rtg2::LEU2 *	This study
ZLY2570	BY4742 *rtg3::URA3 *	This study
ZLY2648	BY4741 *rtg1::LEU2 aco1::kanMX4 *	This study
ZLY2631	BY4741 *rtg2::LEU2 aco1::kanMX4 *	This study
ZLY2652	BY4741 *rtg3::URA3 aco1::kanMX4 *	This study
ZLY3206	*MAT*α* met? [rho* ^ 0^ *] *	This study
CS725-3A	*MATa ura3 leu2 his3 met15 cit1/2/3::kanMX4 *	[21]
ZLY2545	CS725-3A *aco1::HIS3 *	[21]
RBY353	BY4741 *cit1::kanMX4 *	Research genetics
RBY355	BY4741 *cit2::kanMX4 *	Research genetics
RBY356	BY4741 *cit3::kanMX4 *	Research genetics
ZLY2603	*MATa ura3 leu2 met15 aco1::kanMX4 *	This study
RBY469	BY4741 *cit1::kanMX4 aco1::kanMX4 *	This study
RBY277	BY4741 *cit2::kanMX4 aco1::kanMX4 *	This study
RBY363	BY4741 *cit3::kanMX4 aco1::kanMX4 *	This study
ZLY854	BY4741 *cit1/2::kanMX4 aco1::kanMX4 *	This study
BY4741 (*mrs3 mrs4*)	BY4741 *mrs3::kanMX4 mrs4::kanMX4 *	[29]
ZLY3505	BY4741 *mrs3::kanMX4 mrs4::kanMX4 aco1::HIS3 *	This study
ZLY4603	BY4741 *sod1::kanMX4 *	This study
BY4741 (*sod2*)	BY4741 *sod2::kanMX4 *	Research genetics
TPY1507	BY4741 *sod1::kanMX4 aco1::kanMX4 *	This study
ZLY3973	BY4741 *sod2::kanMX4 aco1::kanMX4 *	This study

**Table 2:** Plasmids used in this study.

Plasmid	Description	Source
pRS416	A yeast centromeric plasmid carrying *URA3* selection marker	[30]
pUC-rtg1::LEU2	An *rtg1::LEU2* disruption cassette in pUC19	[31]
pUC-rtg2::LEU2	An* rtg2::LEU2* disruption cassette in pUC19	[31]
pUC-rtg3::URA3	An *rtg3::URA3* disruption cassette in pUC19	[32]
pBS-aco1::HIS3	An *aco1::HIS3*disruption cassette in pBluescript	This study
pCIT2-lacZ	A *CIT2-lacZ* reporter gene on the plasmid pWCJ (*CEN URA3*)	This study
pRS303-SOD1	The *SOD1* gene was cloned into the integrative plasmid pRS303	This study
